# Estimation of genetic parameters for pork belly traits

**DOI:** 10.5713/ab.22.0391

**Published:** 2023-02-27

**Authors:** Seung-Hoon Lee, Sang-Hoon Lee, Hee-Bok Park, Jun-Mo Kim

**Affiliations:** 1 Department of Animal Science and Technology, Chung-Ang University, Anseong 17546, Korea; 2 Department of Biotechnology, Korea University, Seoul 02841, Korea; 3 Division of Pediatric General and Thoracic Surgery, Cincinnati Children’s Hospital Medical Center, Cincinnati, OH, 45229, USA; 4 Department of Animal Resources, Kongju National University, Yesan 32439, Korea

**Keywords:** Belly Component, Genetic Correlation, Genetic Parameter, Heritability, Pig, Pork Belly

## Abstract

**Objective:**

Pork belly is a cut of meat with high worldwide demand. However, although the belly is comprised of multiple muscles and fat, unlike the loin muscle, research on their genetic parameters has yet to focus on a representative cut. To use swine breeding, it is necessary to estimate heritability against pork belly traits. Moreover, estimating genetic correlations is needed to identify genetic relationship among the traditional carcass and meat quality traits. This study sought to estimate the heritability of the carcass, belly, and their component traits, as well as the genetic correlations among them, to confirm whether these traits can be improved.

**Methods:**

A total of 543 Yorkshire pigs (406 castrated males and 137 females) from 49 sires and 244 dam were used in this study. To estimate genetic parameters, a total of 12 traits such as lean meat production ability, meat quality and pork belly traits were chosen. The heritabilities were estimated by using genome-wide efficient mixed model association software. The statistical model was selected so that farm, carcass weight, sex, and slaughter season were fixed effects. In addition, its genetic parameters were calculated via MTG2 software.

**Results:**

The heritability estimates for the 7th belly slice along the whole plate and its components were low to moderate (0.07±0.07 to 0.33±0.07). Moreover, the genetic correlations among the carcass and belly traits were moderate to high (0.28±0.20 to 0.99±0.31). Particularly, the rectus abdominis muscle exhibited a high absolute genetic correlation with the belly and meat quality (0.73±52 to 0.93±0.43).

**Conclusion:**

A moderate to high correlation coefficient was obtained based on the genetic parameters. The belly could be genetically improved to contain a larger proportion of muscle regardless of lean meat production ability.

## INTRODUCTION

Pork is one of the most consumed meats in the world and constitutes a major source of animal protein, especially in Asian countries such as South Korea [1,2]. Moreover, South Korean consumers show a specific preference for the belly, which is mainly consumed as a roasting cook and is known to be a high-fat cut [3–5]. Pork belly has also become one of the most valuable consumed meats in the western world [6,7]. However, the yield of the belly meat is negatively correlated with its lean content [6,8].

Among other factors, pig breeding primarily aims to improve lean meat production and carcass yield. Therefore, current systems to evaluate pig breeding performance have mainly focused on the longissimus thoracis (LT) muscle, which corresponds to the loin eye area (LEA). However, focusing on improving the lean percentage of the carcass has the unintended consequence of reducing the pork belly percentage [8,9]. Therefore, this LT-based assessment can be problematic in regions of the world where consumers prefer pork belly. To solve this problem, a new evaluation system for pork belly must be established. In fact, a previous study suggested that pork belly should become an independent evaluation item [10].

Unlike the LT, which is made up of a single muscle, pork belly is comprised of multiple muscles with fat content between them [7]. Therefore, an appropriate understanding of the muscles that comprise the pork belly should be established to favor the production of animals with larger and leaner bellies. Nevertheless, there are currently no standards regarding the slices or component muscles of pork belly to serve as a reference for animal breeding. For this reason, a pork belly cut standard was established to improve pork belly quality by analyzing the ratio between muscle and fat, as well as the characteristics of the muscle and fat components of the pork belly [11]. A belly slice section obtained from the 7th cut along the whole plate (hereinafter referred to as “Section 7”) was suggested as a potential representative slice of pork belly. Standardizing pork belly slices would allow for genetic improvement based on phenotypic correlation with the volume of whole belly [11]. Nevertheless, to improve pork belly quality through selective breeding, additional studies are needed to confirm the genetic parameters and correlations among the component muscles from a representative slice from the pork belly and other economic and meat quality traits.

Therefore, our study sought to improve pork belly traits that are directly associated with consumer preference by collecting the characteristics of the belly of the pig, the carcass, and the meat quality in a purebred Yorkshire pig population. Normally, crossbred pigs were used for commercial. However, it needs to estimate the genetic parameters of each purebred to use in swine breeding for pork belly. This study was conducted to estimate genetic parameters, including heritability of the detailed belly features of representative cuts, lean meat production, and meat quality traits, and their genetic correlations. Previous studies have analyzed pork belly parameters but were limited to belly weight (BEW), width, and length [12,13]. Moreover, these parameters did not account for the muscle composition of the pork belly. Therefore, the genetic parameters elucidated in this study may facilitate the selection of desirable pork belly characteristics.

## MATERIALS AND METHODS

This study followed the guidelines of the Institutional Animal Care and Use Committee of the National Institute of Animal Science, Korea (2015-137). This study was conducted from February 2016 to June 2017.

### Animals and muscle samples

In total, 543 Yorkshire pigs (406 castrated males and 137 females) from 49 sire and 244 dam were used in this study. The pedigree information presented in Supplementary Table S1. The animals were born from a single multiplier (grand parents farm, GP) from July 2015 to November 2016 that was a closed population and transported to nine breeding stock farms, which mimicked the original conditions including feeding conditions based on the one breeding company as much as possible. The pigs were slaughtered by 25 times (a total of 25 contemporary groups) in a commercial abattoir following standard procedures under the supervision of the Korean Animal Products Grading Service.

### Carcass trait measurements

The carcass weight (CW) and backfat thickness (BFT) were measured immediately after slaughter. The BFT was measured with a ruler at the 11th and last thoracic vertebrae on the left half of each carcass, and the average of the two measurements was used for the analysis. The LEA was measured at the level of the last rib after chilling the carcasses for 24 h.

### Measurement of belly characteristics

In this study, we measured and estimated multiple pork belly characteristics using a set of pork belly slices. The carcasses were cooled at 4°C for 24 h. Then, the left side of each carcass was divided into seven primal cuts following the standard cutting lines of the Korea Institute for Animal Products Quality Evaluation, and the belly was separated from the shoulder between the 4th and 5th thoracic vertebrae with a straight cut perpendicular to the axis of the carcass. The BEW was measured before dividing the belly into vertical slices.

The belly was cut using a meat cutter (KSC-330Q; Fujee, Siheung, Korea) at every vertebra (6th to 14th thoracic vertebra and 1st to 5th lumbar vertebra), resulting in 14 cuts with a thickness of approximately 3 cm each. Slice numbers were labelled from 1 to 14, from cranial to caudal. Each of the resulting pork belly slices was scanned (DocuPrint, C3360; Canon, Tokyo, Japan) with a steel ruler for calibration. The area (cm^2^) of the belly muscle components in each section was recorded through image scanning using the Image-Pro Plus software (Media Cybernetics, Rockville, MD, USA). The belly area (BA) and muscle area (MA) were then measured for each image slice of the pork bellies. The fat area was calculated by subtracting the MA from the BA. The volume of the belly (VB), belly muscle (VTM), and belly fat (VTF) were computed by summing all the corresponding slice values and multiplying them by the slice thickness. The belly muscle ratio (BMR) and belly fat ratio (BFR) were calculated by dividing the VTM or VTF by the VB and multiplying the value by 100 to convert it to a percentage.

### Measurement of the belly components in Section 7

**Figure 1:**
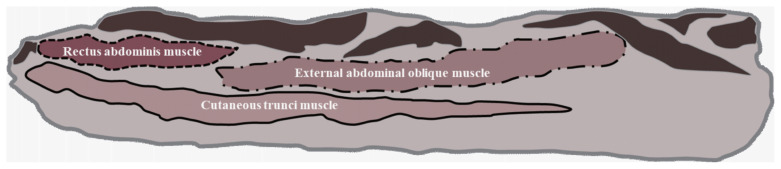
Scheme of belly components in the Section 7 region. The solid line area represents the cutaneous trunci muscle (CTM), the dotted line area indicates the rectus abdominis muscle (RAM), and the dotted and solid mixed line areas represent the external abdominal oblique muscle (EAM).

The definition of the belly component in Section 7 has been previously reported by Lee et al [11]. The three measured muscles that comprise the pork belly in Section 7 and were recorded using Image-Pro Plus software were the cutaneous trunci muscles (CTM), rectus abdominis muscles (RAM), and external abdominal oblique muscles (EAM; Figure 1).

### Meat quality measurements

The pH at 45 min post-mortem (pH45) was measured at the 13th to 14th ribs using a spear-type electrode (Model 290A; Orion Research Inc., Waltham, MA, USA), which was previously calibrated with pH4 and pH7 standard solutions with automatic temperature compensation. The carcasses were chilled at 4°C for 24 h and the LT muscle was obtained to evaluate the meat quality traits. Additionally, samples were cut from the pork loin at the 8th to 9th thoracic vertebrae at 24 h post-mortem and placed on a table for 30 min to expose their surfaces to the air prior to measuring the meat color. The average lightness (L) was recorded using a chromometer (CR-300; Minolta Camera Co., Osaka, Japan), and the results were expressed as CIE (Commission Internationale de l’Eclairage) lightness [14]. The CR-300 chromometer featured an 8 mm open cone aperture. All measurements were conducted under standard illumination (illuminant C) and the standard observer position was 2°. The drip loss (DL) of the loin was determined as described by Honikel [15]. The samples were suspended in an inflated bag at 4°C for 48 h, then weighed after being gently blotted dry. The DL measurements were expressed as a percentage of the initial sample weight.

### Genotypes

The genomic DNA of the experimental animals was isolated from belly muscle tissue using the DNeasy Blood & Tissue Kit (Qiagen, Hilden, Germany). The animals were genotyped for 55,232 single nucleotide polymorphism (SNP) markers using the Axiom Porcine Breeders Genotyping Array (Thermo Fisher, Waltham, MA, USA). Quality control and filtration of genotyped SNP markers were performed using the PLINK software (ver. 1.90) [16]. The genotypes were filtered according to minor allele frequency (<5%), genotype call rate (<90%), and p-value of χ^2^-test for Hardy-Weinberg equilibrium errors (<0.000001). A total of 42,399 SNP markers on 18 autosomes were left after filtration and quality control.

### Statistical analysis

Prior to the statistical analyses, we calculated the descriptive statistics and validated the normal distribution of the phenotype data. Putative outliers were excluded based on the ascertainment of normality using the Shapiro-Wilk’s method in SAS 9.4 (SAS Inst. Inc., Cary, NC, USA).

The heritability for all the traits were estimated for 543 Yorkshire pigs using the univariate linear mixed model (LMM) method in the genome-wide efficient mixed model association (GEMMA) software [17]. The LMM method equation was given by:


(1) y=Xb+Zu=e

where **y** is the phenotype, **b** is the vector of fixed effects including sex, farm, season (summer or non-summer based on month slaughtered), and CW; **u** is the vector of random additive genetic effects following a normal distribution $u\sim \text{N}(0,G\sigma_{u}^{2})$, in which **G** is the n×n genomic relationship matrix (GRM) of which matrix elements are composed of pairwise genetic relationship coefficients computed using genotypes of 42,399 SNP markers, and $\sigma_{u}^{2}$ is the additive genetic variance component; e is the vector of random residuals following a normal distribution $e\sim \text{N}(0,I\sigma_{e}^{2})$, in which I is the n×n identity matrix and $\sigma_{e}^{2}$is the residual variance component. The GEMMA software was used for the construction of the GRM and the estimation of the two variance components (i.e., $\sigma_{u}^{2},\;\sigma_{e}^{2}$) for each univariate trait based on the restricted maximum likelihood method (REML). **X** and **Z** are the incidence matrices for **b** and **u**.

Heritability was estimated as follows:


(2) $$h^{2}=\frac{\sigma_{u}^{2}}{\sigma_{u}^{2}+\sigma_{e}^{2}}$$

Moreover, the outcomes of this estimation were classified as high (*h*^2^≥0.4), moderate (0.2≤*h*^2^≤0.4), or weak (*h*^2^<0.2).

Bivariate analyses were applied to all possible combinations of traits to estimate genetic correlations using the bivariate linear mixed-effects model, as follows:


(3) $$\left[\begin{array}{c} y_{1}\\ y_{2} \end{array}\right]=\left[\begin{array}{cc} X_{1} & 0\\ 0 & X_{2} \end{array}\right]\;\left[\begin{array}{c} b_{1}\\ b_{2} \end{array}\right]+\left[\begin{array}{cc} Z_{1} & 0\\ 0 & Z_{2} \end{array}\right]\;\left[\begin{array}{c} u_{1}\\ u_{2} \end{array}\right]+\left[\begin{array}{c} e_{1}\\ e_{2} \end{array}\right]$$

where **y_1_** and **y_2_** are the vectors of the measured phenotypes for the two traits under consideration; **b_1_** and **b_2_** are the vectors of the fixed effects for the traits under consideration; **u_1_** and **u_2_** are vectors of the random additive genetic effects for the traits under consideration; **X_1_** and **X_2_** are the incidence matrices relating records of the traits to the fixed effects; **Z_1_** and **Z_2_** are the incidence matrices relating observations with random additive genetic effects; and **e_1_** and **e_2_** are the vectors of random residuals. The expectation and variance of the bivariate linear mixed model were as follows:


(4) $$\text{E }\left[\begin{array}{c} y_{1}\\ y_{2} \end{array}\right]=\left[\begin{array}{cc} X_{1} & 0\\ 0 & X_{2} \end{array}\right]\;\left[\begin{array}{c} b_{1}\\ b_{2} \end{array}\right]$$

And


(5) $$\text{Var}\;\left[\begin{array}{c} u_{1}\\ u_{2}\\ e_{1}\\ e_{2} \end{array}\right]=\left[\begin{array}{llll} G\sigma_{u11}^{2} & G\sigma_{\text{u}12}^{2} & 0 & 0\\ G\sigma_{\text{u}21}^{2} & G\sigma_{\text{u}22}^{2} & 0 & 0\\ 0 & 0 & I\sigma_{\text{e}11}^{2} & I\sigma_{\text{e}12}^{2}\\ 0 & 0 & I\sigma_{\text{e}21}^{2} & I\sigma_{\text{e}22}^{2} \end{array}\right]$$

where **G** is the GRM; $\sigma_{\text{u}11}^{2},\;\sigma_{\text{u}12}^{2},\sigma_{\text{u}21}^{2}$, and $\sigma_{\text{u}22}^{2}$ are additive genetic (co)variances for the traits under consideration; $\sigma_{\text{e}11}^{2},\;\sigma_{\text{e}12}^{2},\sigma_{\text{e}21}^{2}$, and $\sigma_{\text{e}22}^{2}$ are residual (co)variances for the traits; **I** is the identity matrix. The vectors **u** and **e** were assumed to be normally distributed, with means and (co)variances equal to 0. All genetic parameters were computed using the MTG2 software, which is based on genomic REML analysis [18]. The correlation between the phenotypes was estimated using Pearson’s method in SAS 9.4. The absolute correlation coefficient estimates (*r*) were classified as strong (*r*≥0.7), moderate (0.3≤*r*<0.7), or weak (*r*<0.3).

## RESULTS

### Descriptive statistics

The descriptive statistics are summarized in Supplementary Table S2. The mean CW was 87.37±9.13 kg. The means of the other carcass traits were 38.22±5.54 cm^2^ (LEA) and 21.95± 5.13 mm (BFT).

The means of the six belly traits were 7.05±1.00 kg for the BEW, 5,954.81±688.54 cm3 for the VB, 2,758.04±381.16 cm^3^ for the VTM, 3,107.01±597.80 cm^3^ for the VTF, 46.50%± 6.54% for the BMR, and 53.50%±6.54% for the BFR. Regarding the belly components in Section 7, the CTM area was 14.96±2.62 cm^2^, the RAM area was 7.07±1.55 cm^2^, and the EAM area was 10.87±2.17 cm^2^. For meat quality, the mean pH45 was 6.13±0.28, the mean L was 46.34±2.75, and the mean DL was 2.69%±1.38%.

### Heritability

The estimated heritability values are summarized in Table 1. For the carcass traits, the CW had a moderate heritability (*h*^2^ = 0.21±0.08), the LEA had the highest heritability values compared to those of the other two carcass traits (*h*^2^ = 0.47± 0.08), and the BFT showed moderate heritability (*h*^2^ = 0.36± 0.07).

The heritability of the belly trait BEW was weak (*h*^2^ = 0.15 ±0.07), whereas the VTF had the highest heritability of all the traits (*h*^2^ = 0.33±0.07). The belly traits VB, VTM, BMR, and BMF exhibited heritability values of 0.21±0.08, 0.19±0.08, 0.25±0.08, and 0.25±0.08, respectively.

For the belly muscle components in Section 7, the heritability of the CTM was 0.23±0.07, and that of the EAM was 0.19±0.06. The RAM had the lowest heritability (*h*^2^ = 0.07 ±0.07). In terms of meat quality, the *h*^2^ of pH45 was 0.09±0.05, and that of L was 0.24±0.07. Furthermore, the estimated heritability of DL was 0.36±0.08.

### Phenotypic correlations

**Figure 2:**
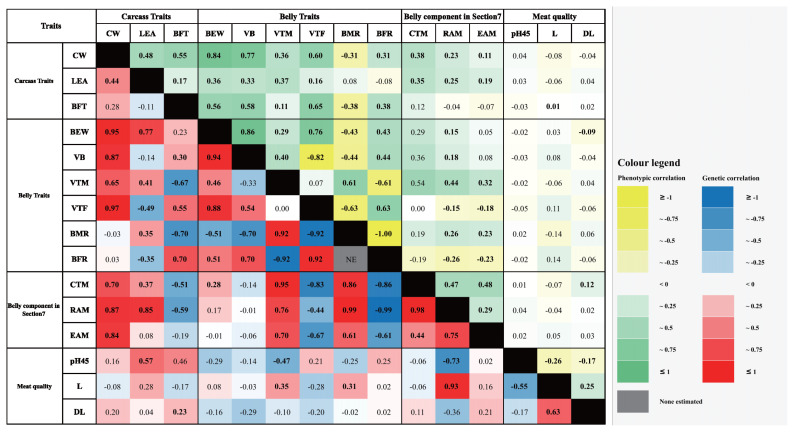
Heat map showing phenotypic and genetic correlation. The color scale bar from yellow to green represents the level of correlation for each phenotype. The color scale bar from blue to red represents the level of correlation between the genetic factors. The grey color indicates no data. The black color indicates blank.

The estimated phenotypic correlations among traits are shown in Figure 2 and Supplementary Table S3. For the carcass traits, the CW had slightly positive phenotypic correlations among the carcass, belly, and belly components in Section 7 (LEA, 0.48; BFT, 0.55; BEW, 0.84; VB, 0.77; VTM, 0.36; VTF, 0.60; BFR, 0.31; CTM, 0.38; RAM, 0.23; and EAM, 0.11). However, the phenotypic correlation between the belly traits CW and BMR had a moderate negative correlation (*r* = −0.31). Moreover, there was no correlation between the CW and meat quality traits. The LEA showed no correlation with the BMR, BFR, and meat quality traits (p>0.05). The LEA had weak correlations with BFT, VTF, RAM, and EAM (0.17, 0.16, 0.25, and 0.19, respectively). The other traits had moderate positive correlations with the LEA (BEW, 0.36, VB, 0.33; VTM, 0.37; and CTM, 0.35). Positive phenotypic correlations were observed between the BFT and several traits, including the BEW, VB, VTM, VTF, BFR, CTM, and L (0.56, 0.58, 0.11, 0.65, 0.38, 0.12, and 0.01, respectively). BMR, one of the muscle-related traits, had a negative correlation coefficient with the BFT (−0.38). The other traits had no correlation with the BFT.

Among the belly traits, BEW exhibited a moderate to high absolute phenotypic correlation coefficient with some belly traits including the VB, VTF, BMR, and BFR (0.86, 0.76, −0.43, and 0.43, respectively). Moreover, the BEW had a weak absolute correlation with VTM, CTM, RAM, and DL (0.29, 0.29, 0.15, and −0.09, respectively). However, the other traits seemed to have a high significance. The VB trait was positively correlated with almost all the belly and belly component traits (VTM, 0.40; BFR, 0.44; CTM, 0.36; and RAM, 0.18), whereas other traits such as VTF and BMR had negative correlations (−0.82 and −0.44, respectively), and three meat quality traits had no correlations with high p-value. The VTM had no correlation with the VTF. Nevertheless, some of the belly and belly component traits without the BFR (−0.61) had moderate positive correlations (BMR, 0.61; CTM, 0.54; RAM, 0.44; and EAM, 0.32). The other traits showed no significance with the low absolute correlation coefficient. The VTF trait had a negative correlation with the belly muscle-associated traits, including the BMR, RAM, and EAM (−0.63, −0.15, and −0.18, respectively). VTF and BFR had a positive correlation coefficient (0.63), whereas the meat quality traits without L had no correlation with the VTF (L, 0.11). The belly trait BMR had a low positive correlation with the belly association traits (CTM, 0.19; RAM, 0.26; and EAM, 0.23). Additionally, L had a weakly negative correlation (−0.14). The BFR was negatively correlated with all three belly components in Section 7 (CTM, −0.19; RAM, −0.26; and EAM, −0.23), and the meat quality trait L had a weakly positive correlation (0.14). However, the correlation of BFR with pH45 and DL seemed to no significance.

For the belly components in Section 7, all traits had weak to moderate positive correlation coefficients (0.47 for CTM with RAM, 0.48 for CTM with EAM, and 0.29 for RAM with EAM). The CTM trait had a weakly positive correlation with the meat quality trait DL (0.12). RAM had no correlation with the meat quality traits. Similarly, no correlations were identified between EAM and the meat quality traits. For the meat quality traits, pH45 had a weak negative correlation with the other traits (L, −0.26 and DL, −0.17). The L trait had a weak positive correlation with DL (0.25).

### Genetic correlations

The genetic correlations among the traits were estimated and are presented with the phenotypic correlations in Figure 2 and Supplementary Table S3. The CW, one of the carcass traits, had a strongly positive genetic correlation with the BEW, VB, CTM, RAM, and EAM (0.95±0.06, 0.87±0.09, 0.70±0.19, 0.87±0.29 and 0.84±0.26, respectively). Additionally, moderate absolute correlations with other traits occurred (VTM, 0.65±0.23 and VTF, 0.37±0.28). However, the other traits had standard errors that were higher than the absolute correlation coefficients. Eight traits had moderate to high absolute correlation coefficients with the LEA. Particularly, the VTF and BFR belly traits had negative correlations (−0.49±0.18, and −0.35±0.21, respectively), whereas the muscle associated traits including the belly trait BEW, VTM, and BMR, the belly components CTM and RAM in Section 7, and pH45 had positive correlations (0.77±0.10, 0.41±0.23, 0.35±0.21, 0.37±0.19, 0.85±0.36, and 0.57±0.52, respectively). The other traits and LEA had weak to moderate absolute correlation coefficients, but those traits showed a higher standard error than their coefficients. The BFT showed a positive correlation with the three belly traits. Particularly, we observed a strong positive correlation between the BFT and BFR (0.70±0.16). Additionally, we observed a positive correlation of 0.30±0.27 for the VB, 0.55±0.16 for the VTF, and 0.23±0.52 for pH45. The other belly traits and belly components in Section 7 exhibited a negative correlation (VTM, −0.67±0.20; BMR, −0.70±0.16; CTM, −0.51±0.18; and RAM, −0.59±0.31). However, BFT did not appear to have a notable correlation with the other traits due to high standard errors.

For the belly traits, the BEW had a moderate to strong absolute correlation coefficient for almost every belly trait (VB, 0.94±0.04; VTM, 0.46±0.20; VTF, 0.88±0.77; BMR, −0.51± 0.19; and BFR, 0.51±0.19). The CTM was the only trait of the belly components in Section 7 that was positively correlated with BEW (0.28±0.20). The other traits had a poor to moderate correlation coefficient with the BEW, but they also had a higher standard error than their absolute coefficients. Positive correlations with the VB were found among the belly and belly components in Section 7. The correlations between VB and the VTF and BFR traits were above moderately positive (0.54±0.19, and 0.70±0.25, respectively). Moreover, VTM had above moderately positive correlation with the belly trait BMR and the belly components CTM, RAM, and EAM (0.92±0.05, 0.95±0.10, 0.76±0.18, and 0.70±0.23, respectively). Additionally, the VTM was positively correlated with the meat quality trait L (0.35±0.26). BFR (another belly trait) and the meat quality traits pH45 and DL had negative correlations with the meat quality trait L (BFR, −0.92±0.05; pH45, −0.47±0.42, and DL, −0.10±0.27). The standard errors of the correlation between the VTM and the VTF were higher than their absolute correlation coefficients. The VTM had a moderate to high absolute correlation with the belly traits and the belly components in Section 7 (BMR, −0.92±0.09; BFR, 0.92 ±0.09; CTM, −0.83±0.14; RAM, −0.44±0.21; and EAM, −0.67 ±0.19). The BMR trait was above moderately positively correlated with the CTM, RAM, EAM, and L (0.86±0.16, 0.99 ±0.31, 0.61±0.21, and 0.31±0.24, respectively). On the other hand, there was no correlation between the BMR and the other traits. Negative genetic correlations with the BFR were found among three of the belly components in Section 7 (CTM, −0.86±0.16; RAM, −0.99 ±0.31; and EAM, −0.61± 0.21). However, the BFR had no correlation with the meat quality traits.

The CTM of the belly component in Section 7 and the RAM appeared to have a strong positive correlation coefficient (0.98±0.34), and the correlation coefficient between the CTM and EAM was moderately positive (0.44±0.21). The RAM also showed a strong positive correlation with the EAM (0.75±0.49). Therefore, RAM and pH45 exhibited a strong absolute correlation with the meat quality trait L (−0.73 ±0.52 and 0.93±0.43, respectively). The EAM had no correlation with the meat quality traits.

The meat quality parameter pH45 had a negative correlation with L (−0.55±0.35). Moreover, the standard error of the correlation coefficient between pH45 and DL was higher than the absolute coefficient, and L had a moderately positively correlation with DL (0.63±0.18).

## DISCUSSION

Pork belly is among the most demanded cuts in South Korea [3,4,19] and other countries worldwide, including the East Asia region. Previous studies have assessed the heritability and genetic parameters of pork belly traits [20,21]. However, these studies did not estimate these parameters for the representative traits of pork belly. Previously, Lee et al. defined the representative sections of the belly and their parameters [11]. In this study, Section 7 of the belly was suggested as the best section for belly assessment based on the MA in each vertebra [11]. Our study sought to estimate the correlations between genetic parameters associated with swine carcass traits, belly traits, and belly components in Section 7, and the meat quality in Yorkshire pigs. We first estimated the genetic parameters for the belly components.

The estimated heritability values for the carcass traits in this study ranged from 0.21 to 0.47. A previous study demonstrated that the heritability of the carcass traits CW and BFT of Yorkshire pigs were 0.21 and 0.54, respectively [22]. These results were similar to our heritability results for the CW and BFT. A previous study indicated that the heritability of the LEA was moderate (*h*^2^ = 0.28) in Yorkshire pigs [23]. However, another study in the US that estimated the heritability of the LEA found a high heritability (*h*^2^ = 0.56) in Yorkshire pigs [24]. In other words, these two studies reached different conclusions regarding the heritability of the LEA trait for the same breed with similar litter sizes. However, this discrepancy was likely due to differences in the estimation methods. The results of the present study also differed from those of these other studies. This difference was likely caused by variations in litter size and other environmental factors.

For the belly traits, the estimated heritability ranged from 0.15 to 0.42. Kang et al [21] previously reported that the BEW had a moderate heritability compared to our estimates (*h*^2^ = 0.33). Additionally, another study in South Korea previously reported a heritability of 0.44 [25]. Moreover, Newcom et al [26] reported a BEW heritability of 0.51 in the Yorkshire and purebred Duroc [26]. However, in this study, the heritability of the BEW was 0.15. These results consider that BEW is affected by CW because of the BEW which part of the CW. To avoid overestimation, CW included as a covariate factor for estimating heritability instead of plant age. Therefore, the low heritability against BEW was differently estimated with the other heritability studies. Normally, the commercial pigs were used crossbred (i.e., Yorkshire×Landrace ×Duroc and Landrace×Berkshire×Duroc crossbred). Therefore, we suggest that the low heritability could be supplemented by the other breeds’ heritability. The belly volume traits (i.e., VB, VTM, and VTF) were estimated as having weak to moderate heritability in the present study. Other previous studies did not estimate the heritability of the volume traits in pork belly [21]. However, the method used to calculate the volume allowed for the estimation of these traits based on the area traits of the belly. Kang et al [21] also estimated the heritability of pork belly areas, including the muscle and fat. Their estimated heritability values for the muscle and fat area were 0.45 and 0.27, respectively. These results were consistent with those of our study, suggesting that volume traits could be moderately affected by genetic parameters. Moreover, their moderate heritability could be useful for improving the belly volume. However, the heritability of the BMR and BFR, which were estimated in this study, were not reported previously. Many studies have reported the heritability of traits that favor a lean carcass percentage. For example, Lundeheim et al [27] reported that the lean percentage had a high heritability (*h*^2^ = 0.67) [27]. Another study on the Duroc breed estimated that the heritability of lean percentage was 0.73 [28]. Additionally, other studies have also reported a moderate to high heritability of the lean percentage [29]. Therefore, the lean meat production trait is considered to be a highly heritable trait in swine, whereas the heritability of the belly muscle percentage was estimated as moderate in our study. This moderate heritability can be improved by selection and mating [30]. Therefore, the muscle ratio in the pork belly could be enhanced. These findings indicated that belly muscle and fat could be affected by other factors in addition to genetics. For instance, variations could be attributed to environmental changes such as seasonality or moving stress in the finishing stage, as the animals used in this study were moved from the GP to normal farms.

The heritability of the belly components of Section 7 were low to moderate in the present study. In a previous study, Section 7 was chosen as the representative parameter for the belly [11]. Interestingly, the authors suggested that the CTM and RAM could play a key role in the estimation of belly MA [11]. In the present study, the estimated heritability of the aforementioned traits was moderate and weak, respectively. This result is consistent with previous research that estimated the heritability of carcass traits [31]. However, the heritability of the RAM was estimated to be low. These results are consistent with those of Kang et al [21], who estimated the heritability of pork belly components [21]. Nevertheless, the three component muscles including CTM, RAM, and EAM were observed in all slices of the pork belly. Moreover, Lee et al [11] previously reported that those components had a positive phenotypic correlation with VB. Therefore, increasing these components could improve pork belly quality by reducing excess fat, thus enhancing consumer preference. Although the estimated heritability of all three pork belly components in this study did not appear high, this could be overcome by identifying associated genes and their regulatory factors. Therefore, further studies with larger population sizes would improve estimation accuracy. Furthermore, additional studies are needed to identify genes associated with pork belly parameters and assess their applicability to pig breeding.

The present study also estimated the heritability of meat quality traits as low to moderate in Yorkshire pigs. A previous study reported that meat quality traits had a low heritability in Jeju black pigs [32]. The results of this study broadly supported these earlier findings. Moreover, previous studies have reported that the heritability of meat quality traits is low to moderate [30,33,34]. These results, including those of the present study, indicate that meat quality parameters such as pH are likely affected by environmental factors.

In this study, we also estimated the phenotypic correlations among the carcass, belly, and belly components in Section 7 and the meat quality traits. The correlation coefficients between the carcass and meat quality traits were estimated to be low. Low correlations were also previously reported in other studies [32,35]. However, these studies estimated lean meat production ability, which is represented by carcass traits with muscle fiber characteristics. Other studies have estimated the phenotypic correlation between the carcass and meat quality traits and reported a low correlation coefficient or no significance [36,37]. These results indicate that the carcass and meat quality traits have a low phenotypic correlation. The phenotypic correlations among the carcass traits, belly traits, and belly components in Section 7 had moderate to high correlation coefficients in the present study. Particularly, the CW showed a strong correlation with the BEW and VB, suggesting that BEW and VB are related to the CW. Interestingly, the LEA, which corresponds to the LT muscle, was positively correlated with the muscle-associated traits of the belly and belly components. Moreover, the BFT, which was a fat-associated trait, also showed the same magnitude of correlation as the fat traits. Pork belly consists of various muscles and fat [7], which would explain the aforementioned results. Furthermore, a previous study reported that the CW is related to the BEW [38]. This suggests that the phenotypic correlations between the carcass traits in the present study, which are representative for lean meat production ability [39], and the other traits should be carefully considered when creating breeding strategies to improve the characteristics of pork belly and their related traits.

Other phenotypic correlation results between the belly and belly components in Section 7 in this study showed that the BEW and VB had a higher correlation coefficient with VTF than with VTM. Similar findings were also observed for the BMR and BFR. Fredeen [38] reported that a high BEW was linked to a higher fat ratio compared to a lower BEW. However, consumers prefer a high muscle ratio in the belly [40,41]. On the other hand, pork belly with high muscle ratios tends to be less firm, which is a less desirable trait from the perspective of meat processors [42]. Therefore, to cater to the preferences of consumers, the proportions of soft fatty tissues should be reduced by increasing the proportions of CTM, RAM, and EAM, and not by indiscriminately reducing fat [43]. Based on the results of this study and previous reports, we confirmed that the phenotype with a high BEW and VB could increase the belly fat content. For the belly components in Section 7, the CTM had a correlation with the meat quality trait DL. Previous studies have linked the DL to the muscle fiber composition [44–46]. In the present study, we did not measure the muscle fiber characteristics of the belly muscle components. Based on the previous studies, the CTM considers to contain many type IIb muscle fibers. However, additional studies are needed to characterize the muscle fibers of pork belly to obtain more accurate results.

In the present study, we estimated the genetic correlations among traits, including the belly and belly components. The CW was found to be correlated with the LEA, BEW, VB, VTM, VTF, CTM, RAM, and EAM. Additionally, other carcass traits were genetically related to the belly and belly component traits. The genetic correlation between the CW and LEA is known to be genetically associated [24]. Moreover, another study reported that the CW and BEW are also strongly genetically related and positively correlated [25]. Furthermore, pigs have been genetically bred to improve their economic traits [30]. According to traditional breeding strategies, economic traits (i.e., CW, BFT, and LEA) have been improved and their related traits have been phenotypically increased [25,33]. However, these genetic enhancements could increase the fat ratio in the belly, thus decreasing consumer acceptability. In the present study, we reported that the CW, a representative economic trait, showed a genetically positive correlation with the belly components. Furthermore, the CTM and RAM areas were genetically reduced as the BFT increased. Therefore, these results indicate that the belly component traits could be improved without affecting the conventional carcass traits.

Among the genetic correlations in the belly and belly components in Section 7, we observed that the BEW had a positive correlation with the VB, VTM, VTF, BFR, and CTM, whereas the BMR had a negative correlation. Given that all traits belonged to the same or similar category as the BEW, these traits had to be related. Most importantly, our findings indicated that the fat-related traits (i.e., VTF and BFR) were related to the increase in fat traits according to the increase in the CW or weight, which was consistent with the findings of early studies [38]. In the present study, we observed that the VTM was strongly positively correlated with the belly components. Moreover, the VTM was also genetically related to meat quality traits, thereby increasing L values. According to a previous study, the VTM based on the MA of belly could be affected by the belly component traits [11]. Therefore, our findings suggested that the VTM could be genetically affected by the abdominal component traits.

Interestingly, we observed that the belly component traits were genetically related to economic traits and meat quality. Particularly, from a genetic perspective, the RAM of the belly component traits in Section 7 was strongly positively associated with the meat quality trait L, and negatively related to pH45. As mentioned above, the L trait has been previously linked to type I muscle fiber characteristics [32,45,46]. Therefore, the present study suggests that belly component traits are genetically associated with type I muscle fibers. However, additional studies are needed to genetically improve belly traits and their components to cater to consumer preferences. Particularly, muscle fiber characteristics in the belly components should be studied and their genetic factors should be identified based on previously reported muscle fiber characteristics [32,47,48]. Nevertheless, our estimates indicated that the heritability of the RAM was weaker than that of other belly components in Section 7. Moreover, phenotypic correlation analyses revealed that the heritability of the aforementioned trait was weakly negative, which could be attributed to environmental effects on the pH and L traits. Therefore, further studies are required to estimate additive genetic effects and identify genes or genetic factors to improve these effects.

## CONCLUSION

In this study, we estimated the genetic parameters of pork belly, carcass, and meat quality traits. The heritability among the belly and its components in Section 7 were found to be moderate. These results provide a basis for improving the quality of pork belly and its components to obtain a product with less fat and more muscle. Moreover, the RAM was genetically correlated with carcass traits and meat quality. Additionally, pork belly with less fat are generally less firm, which is an undesirable trait for meat processing. Therefore, only de soft fatty portions should be reduced rather than reducing too much fat. Taken together, our findings suggest that the muscle components in pork belly could be genetically enhanced through selective breeding without affecting the carcass traits. Furthermore, as these traits could be also related to the meat quality traits, it is possible to concurrently improve the meat quality.

## Figures and Tables

**Table 1:** Estimates of the genetic ( $\sigma_{g}^{2}$) and environmental ( $\sigma_{e}^{2}$) variances and heritability (*h*^2^) for the carcass, pork belly, and meat quality traits in Yorkshire pigs

Traits	$\sigma_{g}^{2}$	$\sigma_{e}^{2}$	*h* ^2^
Carcass traits			
CW (kg)^1)^	17.43	65.65	0.21±0.08^2)^
LEA (cm^2^)	13.20	14.78	0.47±0.08
BFT (mm)	8.08	14.39	0.36±0.07
Belly traits			
BEW (kg)	7.59	42.99	0.15±0.07
VB (×1,000 cm^3^)	69.56	258.35	0.21±0.08
VTM (×1,000 cm^3^)	25.80	112.03	0.19±0.08
VTF (×1,000 cm^3^)	92.86	186.71	0.33±0.07
BMR (%)	9.90	29.72	0.25±0.08
BFR (%)	9.90	29.72	0.25±0.08
Belly muscle components in Section 7			
CTM (cm^2^)	1.44	4.83	0.23±0.07
RAM (cm^2^)	0.14	2.03	0.07±0.07
EAM (cm^2^)	0.87	3.68	0.19±0.06
Meat quality			
pH45	0.01	0.07	0.09±0.05
L	1.66	5.14	0.24±0.07
DL (%)	0.71	1.26	0.36±0.08

CW, carcass weight; LEA, loin eye area; BFT, backfat thickness; BEW, belly weight; VB, volume of total belly; VTM, volume of total muscle in belly; VTF, volume of total fat in belly; BMR, belly muscle ratio; BFR, belly fat ratio; belly fat ratio; CTM, cutaneous trunci muscle; RAM, rectus abdominis muscle; EAM, external abdominal oblique muscle; L, lightness; DL, drip loss.

1) To estimate the variance components of CW, the following fixed effects were included in the linear mixed model: intercept, sex, farm, season and plant age.

2) Standard error.

## SUPPLEMENTARY MATERIAL

**Supplementary Table S1.** Pedigree information

**Supplementary Table S2.** Descriptive unadjusted statistics for the traits

**Supplementary Table S3.** Genetic (line below) and phenotypic correlation (line above) among the carcass traits, belly traits, and belly components in Section 7, and the meat quality traits
