# Supplementary material for: Estimation of genetic parameters for pork belly traits
Source: Anim Biosci. 2023 Feb 27;36(8):1156–66. doi: 10.5713/ab.22.0391 (PMC10330976; doi:10.5713/ab.22.0391)
Supplement: Supplementary file 1 [file ab-22-0391-Supplementary-Table-1.pdf]

**Table S1.** Pedigree information.

| No | ID | Sire | Dam |
|---|---|---|---|
| 1 | Y001 | S001 | D017 |
| 2 | Y002 | S001 | D175 |
| 3 | Y003 | S001 | D175 |
| 4 | Y004 | S002 | D001 |
| 5 | Y005 | S002 | D001 |
| 6 | Y006 | S002 | D173 |
| 7 | Y007 | S002 | D173 |
| 8 | Y008 | S002 | D174 |
| 9 | Y009 | S003 | D040 |
| 10 | Y010 | S003 | D040 |
| 11 | Y011 | S003 | D040 |
| 12 | Y012 | S003 | D056 |
| 13 | Y013 | S003 | D056 |
| 14 | Y014 | S003 | D056 |
| 15 | Y015 | S003 | D067 |
| 16 | Y016 | S003 | D076 |
| 17 | Y017 | S003 | D076 |
| 18 | Y018 | S003 | D098 |
| 19 | Y019 | S003 | D098 |
| 20 | Y020 | S003 | D098 |
| 21 | Y021 | S004 | D030 |
| 22 | Y022 | S004 | D030 |
| 23 | Y023 | S004 | D107 |
| 24 | Y024 | S004 | D107 |
| 25 | Y025 | S005 | D014 |
| 26 | Y026 | S005 | D018 |
| 27 | Y027 | S005 | D018 |
| 28 | Y028 | S005 | D027 |
| 29 | Y029 | S006 | D007 |
| 30 | Y030 | S006 | D007 |
| 31 | Y031 | S006 | D022 |
| 32 | Y032 | S006 | D022 |
| 33 | Y033 | S006 | D049 |
| 34 | Y034 | S006 | D049 |
| 35 | Y035 | S006 | D062 |
| 36 | Y036 | S006 | D062 |
| 37 | Y037 | S006 | D062 |
| 38 | Y038 | S006 | D091 |
| 39 | Y039 | S006 | D119 |
| 40 | Y040 | S006 | D135 |
| 41 | Y041 | S006 | D135 |

| No | ID | Sire | Dam |
|---|---|---|---|
| 42 | Y042 | S006 | D135 |
| 43 | Y043 | S006 | D153 |
| 44 | Y044 | S007 | D013 |
| 45 | Y045 | S007 | D052 |
| 46 | Y046 | S007 | D080 |
| 47 | Y047 | S007 | D080 |
| 48 | Y048 | S007 | D080 |
| 49 | Y049 | S007 | D136 |
| 50 | Y050 | S007 | D136 |
| 51 | Y051 | S007 | D136 |
| 52 | Y052 | S007 | D136 |
| 53 | Y053 | S007 | D136 |
| 54 | Y054 | S007 | D146 |
| 55 | Y055 | S007 | D146 |
| 56 | Y056 | S007 | D146 |
| 57 | Y057 | S007 | D146 |
| 58 | Y058 | S008 | D054 |
| 59 | Y059 | S008 | D054 |
| 60 | Y060 | S008 | D055 |
| 61 | Y061 | S008 | D055 |
| 62 | Y062 | S008 | D055 |
| 63 | Y063 | S008 | D101 |
| 64 | Y064 | S008 | D131 |
| 65 | Y065 | S008 | D131 |
| 66 | Y066 | S008 | D131 |
| 67 | Y067 | S008 | D131 |
| 68 | Y068 | S008 | D148 |
| 69 | Y069 | S008 | D159 |
| 70 | Y070 | S008 | D159 |
| 71 | Y071 | S008 | D162 |
| 72 | Y072 | S009 | D060 |
| 73 | Y073 | S009 | D139 |
| 74 | Y074 | S009 | D139 |
| 75 | Y075 | S009 | D143 |
| 76 | Y076 | S009 | D143 |
| 77 | Y077 | S009 | D143 |
| 78 | Y078 | S009 | D143 |
| 79 | Y079 | S010 | D004 |
| 80 | Y080 | S010 | D004 |
| 81 | Y081 | S010 | D004 |
| 82 | Y082 | S010 | D011 |
| 83 | Y083 | S010 | D011 |

| No | ID | Sire | Dam |
|---|---|---|---|
| 84 | Y084 | S010 | D020 |
| 85 | Y085 | S010 | D020 |
| 86 | Y086 | S010 | D020 |
| 87 | Y087 | S010 | D021 |
| 88 | Y088 | S010 | D021 |
| 89 | Y089 | S010 | D021 |
| 90 | Y090 | S010 | D039 |
| 91 | Y091 | S010 | D039 |
| 92 | Y092 | S010 | D044 |
| 93 | Y093 | S010 | D044 |
| 94 | Y094 | S010 | D088 |
| 95 | Y095 | S010 | D088 |
| 96 | Y096 | S010 | D088 |
| 97 | Y097 | S010 | D133 |
| 98 | Y098 | S010 | D133 |
| 99 | Y099 | S010 | D137 |
| 100 | Y100 | S010 | D137 |
| 101 | Y101 | S010 | D137 |
| 102 | Y102 | S010 | D150 |
| 103 | Y103 | S010 | D150 |
| 104 | Y104 | S011 | D105 |
| 105 | Y105 | S011 | D105 |
| 106 | Y106 | S011 | D105 |
| 107 | Y107 | S011 | D151 |
| 108 | Y108 | S011 | D151 |
| 109 | Y109 | S011 | D170 |
| 110 | Y110 | S011 | D170 |
| 111 | Y111 | S011 | D170 |
| 112 | Y112 | S011 | D183 |
| 113 | Y113 | S011 | D183 |
| 114 | Y114 | S011 | D183 |
| 115 | Y115 | S012 | D037 |
| 116 | Y116 | S012 | D053 |
| 117 | Y117 | S012 | D053 |
| 118 | Y118 | S012 | D053 |
| 119 | Y119 | S012 | D085 |
| 120 | Y120 | S012 | D085 |
| 121 | Y121 | S012 | D085 |
| 122 | Y122 | S012 | D102 |
| 123 | Y123 | S012 | D130 |
| 124 | Y124 | S012 | D130 |
| 125 | Y125 | S012 | D130 |

| No | ID | Sire | Dam |
|---|---|---|---|
| 126 | Y126 | S012 | D184 |
| 127 | Y127 | S013 | D005 |
| 128 | Y128 | S013 | D005 |
| 129 | Y129 | S013 | D028 |
| 130 | Y130 | S013 | D028 |
| 131 | Y131 | S013 | D048 |
| 132 | Y132 | S013 | D048 |
| 133 | Y133 | S013 | D060 |
| 134 | Y134 | S013 | D060 |
| 135 | Y135 | S013 | D068 |
| 136 | Y136 | S013 | D068 |
| 137 | Y137 | S013 | D068 |
| 138 | Y138 | S013 | D082 |
| 139 | Y139 | S013 | D082 |
| 140 | Y140 | S013 | D082 |
| 141 | Y141 | S013 | D107 |
| 142 | Y142 | S013 | D107 |
| 143 | Y143 | S013 | D107 |
| 144 | Y144 | S013 | D112 |
| 145 | Y145 | S013 | D112 |
| 146 | Y146 | S013 | D126 |
| 147 | Y147 | S013 | D126 |
| 148 | Y148 | S013 | D126 |
| 149 | Y149 | S013 | D155 |
| 150 | Y150 | S013 | D155 |
| 151 | Y151 | S013 | D155 |
| 152 | Y152 | S013 | D155 |
| 153 | Y153 | S013 | D164 |
| 154 | Y154 | S013 | D164 |
| 155 | Y155 | S013 | D164 |
| 156 | Y156 | S013 | D185 |
| 157 | Y157 | S013 | D185 |
| 158 | Y158 | S013 | D185 |
| 159 | Y159 | S014 | D045 |
| 160 | Y160 | S014 | D045 |
| 161 | Y161 | S014 | D047 |
| 162 | Y162 | S014 | D047 |
| 163 | Y163 | S014 | D065 |
| 164 | Y164 | S014 | D065 |
| 165 | Y165 | S014 | D065 |
| 166 | Y166 | S014 | D087 |
| 167 | Y167 | S014 | D087 |

| No | ID | Sire | Dam |
|---|---|---|---|
| 168 | Y168 | S014 | D087 |
| 169 | Y169 | S014 | D087 |
| 170 | Y170 | S014 | D087 |
| 171 | Y171 | S014 | D088 |
| 172 | Y172 | S014 | D088 |
| 173 | Y173 | S014 | D125 |
| 174 | Y174 | S014 | D125 |
| 175 | Y175 | S014 | D127 |
| 176 | Y176 | S014 | D147 |
| 177 | Y177 | S014 | D147 |
| 178 | Y178 | S014 | D165 |
| 179 | Y179 | S014 | D165 |
| 180 | Y180 | S014 | D176 |
| 181 | Y181 | S015 | D009 |
| 182 | Y182 | S015 | D009 |
| 183 | Y183 | S015 | D059 |
| 184 | Y184 | S015 | D059 |
| 185 | Y185 | S015 | D083 |
| 186 | Y186 | S015 | D109 |
| 187 | Y187 | S015 | D109 |
| 188 | Y188 | S015 | D109 |
| 189 | Y189 | S015 | D110 |
| 190 | Y190 | S015 | D110 |
| 191 | Y191 | S015 | D111 |
| 192 | Y192 | S015 | D166 |
| 193 | Y193 | S015 | D166 |
| 194 | Y194 | S015 | D166 |
| 195 | Y195 | S015 | D168 |
| 196 | Y196 | S015 | D168 |
| 197 | Y197 | S015 | D169 |
| 198 | Y198 | S015 | D169 |
| 199 | Y199 | S015 | D169 |
| 200 | Y200 | S016 | D182 |
| 201 | Y201 | S017 | D034 |
| 202 | Y202 | S017 | D034 |
| 203 | Y203 | S017 | D174 |
| 204 | Y204 | S017 | D174 |
| 205 | Y205 | S017 | D193 |
| 206 | Y206 | S017 | D211 |
| 207 | Y207 | S017 | D212 |
| 208 | Y208 | S018 | D002 |
| 209 | Y209 | S018 | D002 |

| No | ID | Sire | Dam |
|---|---|---|---|
| 210 | Y210 | S018 | D002 |
| 211 | Y211 | S018 | D057 |
| 212 | Y212 | S018 | D057 |
| 213 | Y213 | S018 | D075 |
| 214 | Y214 | S018 | D075 |
| 215 | Y215 | S018 | D075 |
| 216 | Y216 | S018 | D075 |
| 217 | Y217 | S018 | D077 |
| 218 | Y218 | S018 | D077 |
| 219 | Y219 | S018 | D078 |
| 220 | Y220 | S018 | D078 |
| 221 | Y221 | S018 | D078 |
| 222 | Y222 | S018 | D124 |
| 223 | Y223 | S018 | D124 |
| 224 | Y224 | S018 | D138 |
| 225 | Y225 | S018 | D138 |
| 226 | Y226 | S018 | D138 |
| 227 | Y227 | S018 | D138 |
| 228 | Y228 | S018 | D145 |
| 229 | Y229 | S018 | D145 |
| 230 | Y230 | S018 | D145 |
| 231 | Y231 | S018 | D145 |
| 232 | Y232 | S018 | D149 |
| 233 | Y233 | S018 | D149 |
| 234 | Y234 | S018 | D149 |
| 235 | Y235 | S018 | D177 |
| 236 | Y236 | S018 | D177 |
| 237 | Y237 | S018 | D177 |
| 238 | Y238 | S018 | D181 |
| 239 | Y239 | S018 | D181 |
| 240 | Y240 | S019 | D012 |
| 241 | Y241 | S019 | D012 |
| 242 | Y242 | S019 | D094 |
| 243 | Y243 | S019 | D094 |
| 244 | Y244 | S019 | D099 |
| 245 | Y245 | S019 | D099 |
| 246 | Y246 | S019 | D099 |
| 247 | Y247 | S019 | D100 |
| 248 | Y248 | S019 | D100 |
| 249 | Y249 | S019 | D108 |
| 250 | Y250 | S019 | D108 |
| 251 | Y251 | S019 | D178 |

| No | ID | Sire | Dam |
|---|---|---|---|
| 252 | Y252 | S019 | D178 |
| 253 | Y253 | S019 | D225 |
| 254 | Y254 | S020 | D024 |
| 255 | Y255 | S020 | D024 |
| 256 | Y256 | S021 | D006 |
| 257 | Y257 | S021 | D023 |
| 258 | Y258 | S021 | D023 |
| 259 | Y259 | S021 | D026 |
| 260 | Y260 | S021 | D026 |
| 261 | Y261 | S021 | D036 |
| 262 | Y262 | S021 | D073 |
| 263 | Y263 | S021 | D073 |
| 264 | Y264 | S021 | D081 |
| 265 | Y265 | S021 | D097 |
| 266 | Y266 | S021 | D097 |
| 267 | Y267 | S021 | D158 |
| 268 | Y268 | S021 | D158 |
| 269 | Y269 | S021 | D158 |
| 270 | Y270 | S022 | D008 |
| 271 | Y271 | S022 | D008 |
| 272 | Y272 | S022 | D016 |
| 273 | Y273 | S022 | D016 |
| 274 | Y274 | S022 | D019 |
| 275 | Y275 | S022 | D066 |
| 276 | Y276 | S022 | D066 |
| 277 | Y277 | S022 | D066 |
| 278 | Y278 | S022 | D074 |
| 279 | Y279 | S022 | D074 |
| 280 | Y280 | S022 | D093 |
| 281 | Y281 | S022 | D093 |
| 282 | Y282 | S022 | D104 |
| 283 | Y283 | S022 | D104 |
| 284 | Y284 | S022 | D128 |
| 285 | Y285 | S022 | D128 |
| 286 | Y286 | S022 | D128 |
| 287 | Y287 | S022 | D180 |
| 288 | Y288 | S022 | D180 |
| 289 | Y289 | S022 | D180 |
| 290 | Y290 | S023 | D173 |
| 291 | Y291 | S023 | D173 |
| 292 | Y292 | S023 | D173 |
| 293 | Y293 | S024 | D043 |

| No | ID | Sire | Dam |
|---|---|---|---|
| 294 | Y294 | S024 | D115 |
| 295 | Y295 | S024 | D115 |
| 296 | Y296 | S024 | D115 |
| 297 | Y297 | S024 | D117 |
| 298 | Y298 | S024 | D123 |
| 299 | Y299 | S024 | D123 |
| 300 | Y300 | S024 | D132 |
| 301 | Y301 | S024 | D132 |
| 302 | Y302 | S024 | D132 |
| 303 | Y303 | S024 | D144 |
| 304 | Y304 | S024 | D144 |
| 305 | Y305 | S024 | D167 |
| 306 | Y306 | S025 | D038 |
| 307 | Y307 | S025 | D038 |
| 308 | Y308 | S025 | D038 |
| 309 | Y309 | S025 | D071 |
| 310 | Y310 | S025 | D071 |
| 311 | Y311 | S025 | D071 |
| 312 | Y312 | S026 | D014 |
| 313 | Y313 | S026 | D029 |
| 314 | Y314 | S026 | D029 |
| 315 | Y315 | S026 | D029 |
| 316 | Y316 | S026 | D035 |
| 317 | Y317 | S026 | D095 |
| 318 | Y318 | S026 | D095 |
| 319 | Y319 | S026 | D142 |
| 320 | Y320 | S026 | D142 |
| 321 | Y321 | S026 | D142 |
| 322 | Y322 | S026 | D154 |
| 323 | Y323 | S026 | D154 |
| 324 | Y324 | S027 | D003 |
| 325 | Y325 | S027 | D003 |
| 326 | Y326 | S027 | D121 |
| 327 | Y327 | S027 | D121 |
| 328 | Y328 | S027 | D121 |
| 329 | Y329 | S027 | D161 |
| 330 | Y330 | S027 | D161 |
| 331 | Y331 | S027 | D161 |
| 332 | Y332 | S027 | D161 |
| 333 | Y333 | S027 | D171 |
| 334 | Y334 | S027 | D171 |
| 335 | Y335 | S028 | D061 |

| No | ID | Sire | Dam |
|---|---|---|---|
| 336 | Y336 | S028 | D061 |
| 337 | Y337 | S028 | D090 |
| 338 | Y338 | S028 | D090 |
| 339 | Y339 | S028 | D090 |
| 340 | Y340 | S028 | D122 |
| 341 | Y341 | S028 | D160 |
| 342 | Y342 | S028 | D197 |
| 343 | Y343 | S028 | D197 |
| 344 | Y344 | S028 | D197 |
| 345 | Y345 | S028 | D204 |
| 346 | Y346 | S028 | D205 |
| 347 | Y347 | S029 | D010 |
| 348 | Y348 | S029 | D010 |
| 349 | Y349 | S029 | D015 |
| 350 | Y350 | S029 | D015 |
| 351 | Y351 | S029 | D015 |
| 352 | Y352 | S029 | D031 |
| 353 | Y353 | S029 | D031 |
| 354 | Y354 | S029 | D031 |
| 355 | Y355 | S029 | D032 |
| 356 | Y356 | S029 | D032 |
| 357 | Y357 | S029 | D032 |
| 358 | Y358 | S029 | D033 |
| 359 | Y359 | S029 | D033 |
| 360 | Y360 | S029 | D033 |
| 361 | Y361 | S029 | D042 |
| 362 | Y362 | S029 | D042 |
| 363 | Y363 | S029 | D050 |
| 364 | Y364 | S029 | D050 |
| 365 | Y365 | S029 | D050 |
| 366 | Y366 | S029 | D069 |
| 367 | Y367 | S029 | D069 |
| 368 | Y368 | S029 | D096 |
| 369 | Y369 | S029 | D096 |
| 370 | Y370 | S029 | D152 |
| 371 | Y371 | S029 | D152 |
| 372 | Y372 | S029 | D152 |
| 373 | Y373 | S030 | D025 |
| 374 | Y374 | S030 | D025 |
| 375 | Y375 | S030 | D064 |
| 376 | Y376 | S030 | D064 |
| 377 | Y377 | S030 | D064 |

| No | ID | Sire | Dam |
|---|---|---|---|
| 378 | Y378 | S030 | D129 |
| 379 | Y379 | S030 | D129 |
| 380 | Y380 | S030 | D129 |
| 381 | Y381 | S030 | D166 |
| 382 | Y382 | S030 | D195 |
| 383 | Y383 | S030 | D199 |
| 384 | Y384 | S030 | D199 |
| 385 | Y385 | S030 | D199 |
| 386 | Y386 | S030 | D199 |
| 387 | Y387 | S030 | D216 |
| 388 | Y388 | S030 | D232 |
| 389 | Y389 | S030 | D232 |
| 390 | Y390 | S030 | D232 |
| 391 | Y391 | S030 | D233 |
| 392 | Y392 | S031 | D084 |
| 393 | Y393 | S031 | D244 |
| 394 | Y394 | S031 | D244 |
| 395 | Y395 | S032 | D106 |
| 396 | Y396 | S032 | D106 |
| 397 | Y397 | S032 | D167 |
| 398 | Y398 | S032 | D167 |
| 399 | Y399 | S032 | D167 |
| 400 | Y400 | S032 | D181 |
| 401 | Y401 | S032 | D190 |
| 402 | Y402 | S032 | D190 |
| 403 | Y403 | S032 | D202 |
| 404 | Y404 | S032 | D208 |
| 405 | Y405 | S032 | D208 |
| 406 | Y406 | S032 | D219 |
| 407 | Y407 | S032 | D219 |
| 408 | Y408 | S032 | D219 |
| 409 | Y409 | S032 | D221 |
| 410 | Y410 | S032 | D221 |
| 411 | Y411 | S032 | D230 |
| 412 | Y412 | S032 | D230 |
| 413 | Y413 | S032 | D230 |
| 414 | Y414 | S032 | D234 |
| 415 | Y415 | S033 | D046 |
| 416 | Y416 | S033 | D114 |
| 417 | Y417 | S033 | D116 |
| 418 | Y418 | S033 | D116 |
| 419 | Y419 | S033 | D206 |

| No | ID | Sire | Dam |
|---|---|---|---|
| 420 | Y420 | S034 | D138 |
| 421 | Y421 | S034 | D138 |
| 422 | Y422 | S034 | D157 |
| 423 | Y423 | S034 | D157 |
| 424 | Y424 | S034 | D198 |
| 425 | Y425 | S034 | D198 |
| 426 | Y426 | S034 | D198 |
| 427 | Y427 | S035 | D091 |
| 428 | Y428 | S035 | D091 |
| 429 | Y429 | S035 | D118 |
| 430 | Y430 | S035 | D188 |
| 431 | Y431 | S035 | D210 |
| 432 | Y432 | S035 | D210 |
| 433 | Y433 | S035 | D210 |
| 434 | Y434 | S035 | D226 |
| 435 | Y435 | S035 | D235 |
| 436 | Y436 | S035 | D238 |
| 437 | Y437 | S036 | D032 |
| 438 | Y438 | S036 | D051 |
| 439 | Y439 | S036 | D068 |
| 440 | Y440 | S036 | D068 |
| 441 | Y441 | S036 | D070 |
| 442 | Y442 | S036 | D086 |
| 443 | Y443 | S036 | D086 |
| 444 | Y444 | S036 | D089 |
| 445 | Y445 | S036 | D100 |
| 446 | Y446 | S036 | D141 |
| 447 | Y447 | S036 | D145 |
| 448 | Y448 | S036 | D145 |
| 449 | Y449 | S036 | D163 |
| 450 | Y450 | S036 | D203 |
| 451 | Y451 | S036 | D207 |
| 452 | Y452 | S036 | D207 |
| 453 | Y453 | S036 | D207 |
| 454 | Y454 | S036 | D228 |
| 455 | Y455 | S037 | D033 |
| 456 | Y456 | S037 | D033 |
| 457 | Y457 | S037 | D072 |
| 458 | Y458 | S037 | D072 |
| 459 | Y459 | S037 | D079 |
| 460 | Y460 | S037 | D079 |
| 461 | Y461 | S037 | D079 |

| No | ID | Sire | Dam |
|---|---|---|---|
| 462 | Y462 | S037 | D134 |
| 463 | Y463 | S037 | D134 |
| 464 | Y464 | S037 | D134 |
| 465 | Y465 | S037 | D172 |
| 466 | Y466 | S037 | D172 |
| 467 | Y467 | S037 | D214 |
| 468 | Y468 | S038 | D092 |
| 469 | Y469 | S038 | D092 |
| 470 | Y470 | S038 | D178 |
| 471 | Y471 | S038 | D178 |
| 472 | Y472 | S038 | D213 |
| 473 | Y473 | S038 | D222 |
| 474 | Y474 | S038 | D222 |
| 475 | Y475 | S039 | D140 |
| 476 | Y476 | S039 | D196 |
| 477 | Y477 | S039 | D231 |
| 478 | Y478 | S039 | D239 |
| 479 | Y479 | S039 | D239 |
| 480 | Y480 | S040 | D002 |
| 481 | Y481 | S040 | D063 |
| 482 | Y482 | S040 | D063 |
| 483 | Y483 | S040 | D063 |
| 484 | Y484 | S040 | D131 |
| 485 | Y485 | S040 | D131 |
| 486 | Y486 | S040 | D131 |
| 487 | Y487 | S040 | D194 |
| 488 | Y488 | S040 | D194 |
| 489 | Y489 | S040 | D194 |
| 490 | Y490 | S040 | D194 |
| 491 | Y491 | S041 | D024 |
| 492 | Y492 | S041 | D209 |
| 493 | Y493 | S041 | D242 |
| 494 | Y494 | S042 | D103 |
| 495 | Y495 | S042 | D186 |
| 496 | Y496 | S042 | D241 |
| 497 | Y497 | S043 | D156 |
| 498 | Y498 | S043 | D156 |
| 499 | Y499 | S043 | D187 |
| 500 | Y500 | S043 | D187 |
| 501 | Y501 | S043 | D191 |
| 502 | Y502 | S043 | D220 |
| 503 | Y503 | S043 | D236 |

| No | ID | Sire | Dam |
|---|---|---|---|
| 504 | Y504 | S043 | D240 |
| 505 | Y505 | S043 | D240 |
| 506 | Y506 | S044 | D058 |
| 507 | Y507 | S044 | D090 |
| 508 | Y508 | S044 | D090 |
| 509 | Y509 | S044 | D097 |
| 510 | Y510 | S044 | D223 |
| 511 | Y511 | S044 | D223 |
| 512 | Y512 | S044 | D227 |
| 513 | Y513 | S044 | D227 |
| 514 | Y514 | S045 | D217 |
| 515 | Y515 | S045 | D217 |
| 516 | Y516 | S046 | D026 |
| 517 | Y517 | S046 | D026 |
| 518 | Y518 | S046 | D179 |
| 519 | Y519 | S046 | D179 |
| 520 | Y520 | S046 | D179 |
| 521 | Y521 | S046 | D218 |
| 522 | Y522 | S046 | D237 |
| 523 | Y523 | S046 | D237 |
| 524 | Y524 | S047 | D041 |
| 525 | Y525 | S047 | D041 |
| 526 | Y526 | S047 | D113 |
| 527 | Y527 | S047 | D113 |
| 528 | Y528 | S047 | D189 |
| 529 | Y529 | S048 | D120 |
| 530 | Y530 | S048 | D183 |
| 531 | Y531 | S048 | D183 |
| 532 | Y532 | S048 | D192 |
| 533 | Y533 | S048 | D200 |
| 534 | Y534 | S048 | D201 |
| 535 | Y535 | S048 | D201 |
| 536 | Y536 | S048 | D224 |
| 537 | Y537 | S049 | D215 |
| 538 | Y538 | S049 | D215 |
| 539 | Y539 | S049 | D229 |
| 540 | Y540 | S049 | D243 |
| 541 | Y541 | S049 | D243 |
| 542 | Y542 | S049 | D243 |
| 543 | Y543 | S049 | D243 |
