# Supplementary material for: Estimation of genetic parameters for pork belly traits
Source: Anim Biosci. 2023 Feb 27;36(8):1156–66. doi: 10.5713/ab.22.0391 (PMC10330976; doi:10.5713/ab.22.0391)
Supplement: Supplementary file 2 [file ab-22-0391-Supplementary-Table-2.pdf]

Table S2. Descriptive unadjusted statistics for the traits.

Traits	N	Mean	SD	Min	Max	S-W
**Carcass Traits**						
Carcass wieght (kg)	540	87.37	9.13	64.00	111.00	0.99
Loin eye area (cm^2)	511	38.22	5.54	21.00	57.00	0.99
Backfat thickness (mm)	542	21.95	5.13	9.00	35.00	0.99
**Belly Traits**						
Belly weight (kg)	529	7.05	1.00	4.50	9.50	0.98
Volume of total belly (cm^3)	535	5954.81	688.54	4126.53	7781.64	0.99
Volume of total belly muscle (cm^3)	543	2758.04	381.16	1709.88	4162.86	1.00
Volume of total belly fat (cm^3)	524	3107.01	597.80	1552.51	4654.30	0.99
Total muslce ratio (%)	543	46.50	6.54	29.88	68.69	0.99
Total Fat ratio (%)	543	53.50	6.54	31.31	70.12	0.99
**Belly musle component in Section7**						
CTM (cm^2)	532	14.96	2.62	8.92	24.02	0.99
RAM (cm^2)	540	7.07	1.55	2.62	11.36	0.99
EAM (cm^2)	526	10.87	2.17	5.19	16.64	0.99
**Meat quality**						
pH45	511	6.13	0.28	5.31	6.94	0.99
Lightness	536	46.34	2.75	37.78	53.26	1.00
Drip loss (%)	513	2.69	1.38	0.34	6.38	0.95
